# Incidence, prevalence, and mortality of sarcoidosis in England: a population-based study

**DOI:** 10.1016/j.lanepe.2025.101283

**Published:** 2025-04-04

**Authors:** Katie Bechman, Mark D. Russell, Kathryn Biddle, Mark Gibson, Maryam Adas, Zijing Yang, Samir Patel, Alex Dregan, Sarah Walsh, Peter Brex, Amit Patel, Katherine J. Myall, Sam Norton, Surinder S. Birring, James Galloway

**Affiliations:** a Centre for Rheumatic Diseases, Department of Inflammation Biology, King's College London, London, UK; b Department of Dermatology, King's College Hospital, London, UK; c Department of Neurology, King's College Hospital, London, UK; d Department of Respiratory Medicine, King's College Hospital, London, UK; e Department of Psychological Medicine, Institute of Psychiatry, Psychology and Neuroscience, King's College London, London, UK; f Centre for Human & Applied Physiological Sciences, School of Basic & Medical Biosciences, Faculty of Life Sciences & Medicine, King's College London, London, UK

**Keywords:** Sarcoidosis, Incidence, Prevalence, Mortality, England

## Abstract

**Background:**

The epidemiology of sarcoidosis in England is largely uncharted, with no population-level prevalence data and outdated incidence and mortality estimates. Our objective was to investigate contemporary trends in incidence, prevalence, and mortality.

**Methods:**

This cohort study used primary care data from the UK Clinical Practice Research Datalink (CPRD), linked to secondary-care and national death registration. Patients aged ≥18 with sarcoidosis were identified using primary care codes. Age-and-sex standardised incidence and prevalence were calculated. Standardised mortality ratios (SMRs) compared mortality with the general population. A matched non-sarcoidosis cohort was constructed within CPRD, and Poisson regression compared all-cause mortality between incident cases and controls.

**Findings:**

Between 2003 and 2023, 18,554 incident sarcoidosis patients were identified. The age- and sex-standardised incidence per 100,000 person-years increased from 6.65 in 2003 to 7.73 in 2023, with the most pronounced rise occurring between 2010 and 2016. Incidence rose notably among males and those over 60-year-olds. Sarcoidosis prevalence increased from 167 to 230 per 100,000 individuals. The age-and-sex standardised all-cause mortality rate was 12.2 per 1000 patients in 2023. Elevated mortality was observed in males [SMR: 1.8 (1.7–1.8)] and females [SMR: 2.1(2.0–2.2)], particularly in those aged 30–70 years old. Regression models indicated higher all-cause mortality in the incident sarcoidosis cohort compared to controls [adjusted mortality rate ratio 1.36 (95% CI 1.27–1.44)].

**Interpretation:**

Sarcoidosis incidence has increased during the study period, with shifts in age-and-sex distribution and excess mortality risk. Recognising this burden is key to refining healthcare policies, optimising resources and improving patient outcomes.

**Funding:**

None.

**Research in context**

**Evidence before this study**

We searched PubMed for reports published from inception to November 2024, that included “sarcoidosis” and “incidence” or “prevalence” or “mortality” or “death” in their title or abstract. We also reviewed references from clinical practice guidelines and global epidemiology studies. Our search yielded publications covering data from various regions worldwide, including Europe (specifically Ireland, Poland, Italy, Spain, Sweden, Denmark, and Switzerland), North America (USA and Canada), Asia (Japan, Korea and Taiwan) and Australia. There were differences in the approach to age-standardisation, rendering comparisons between countries challenging. Data from the UK was limited to a single study based on a relatively small database, analysing data from 1991 to 2003.

**Added value of this study**

This study offers valuable contemporary data on the incidence, prevalence and mortality of sarcoidosis in England, based on a large and representative population. An increasing incidence of sarcoidosis was observed during the study period, particularly among males and individuals over 60 years of age. Both crude and standardised incidence rates are reported, establishing a robust baseline for future international comparisons. All-cause mortality was higher in individuals with sarcoidosis compared to the general population. This elevated mortality risk was also evident in the incident cohort when compared to age-and-sex-matched controls.

**Implications of all the available evidence**

Recognising the increasing burden of sarcoidosis in England, along with shifts in age and sex distribution and increased mortality risk, is crucial for informing healthcare policies and clinical practices. A thorough understanding of these epidemiological trends supports the development of more effective healthcare strategies, optimises resource allocation, and enables targeted interventions for high-risk populations.

## Introduction

Sarcoidosis is a systemic granulomatous disorder of unknown aetiology. Clinical manifestations vary depending on the organ involved. The course is unpredictable, ranging from mild self-limiting disease to a chronic, progressive form with severe organ involvement and premature death.^1^^,^^2^ There is well established global variation in the prevalence of sarcoidosis, with the highest rates reported in the northern hemisphere.^3^, ^4^, ^5^, ^6^ Genetic and environmental factors contributing to disease risk are likely to explain much of this geographical variation. There are also differences between ethnicities^7^^,^^8^ and in age and sex distribution.^3^^,^^8^ Mortality from sarcoidosis is primarily attributable to pulmonary and cardiac complications. Mortality rates vary by age, ethnicity, gender and geographical location^9^, ^10^, ^11^ with evidence suggesting an increasing trend over time.^9^

Despite its clinical significance, the epidemiological landscape of sarcoidosis in England is a blank canvas. There are no population-level data on the prevalence. The incidence was last calculated 20 years ago using data from only 2% of general practices,^12^ with a rate far lower than estimates from Sweden, the USA and Canada.^3^^,^^4^^,^^8^ Data on mortality are also outdated. UK wide studies from 15 years ago report a twofold increase in the risk of death compared with matched controls,^12^ with a rising mortality rate over time.^13^

Understanding the epidemiology of sarcoidosis in England is important for early diagnosis, effective resource allocation, informing public health strategies, and optimising patient care. The scarcity of data has hindered research efforts in this area, which are even more critical now with the growing focus on emerging therapies. To address these knowledge gaps, we performed a population-based study using a longitudinal database of linked primary and secondary care records on millions of individuals,^14^ to assess trends in sarcoidosis incidence, prevalence and mortality.

## Methods

### Data sources

The Clinical Practice Research Datalink (CPRD) Aurum is a UK-based, large-scale primary care database widely used for epidemiological and clinical research.^14^ It contains anonymised, longitudinal electronic health records (EHR) collected from general practices using EMIS Web® health record software. CPRD includes data on approximately 47 million people who are currently or were previously registered with participating practices, with around 16 million patients currently registered, covering 24% of the UK population.^15^ The dataset provides comprehensive patient information, including demographics, diagnoses, prescriptions, referrals, and test results, with data available from 1989 to the present.

As EMIS Web® is one of the most widely used general practice systems in the UK, CPRD offers extensive geographical coverage and a population that is representative of the UK in terms of age, sex, and regional distribution, enhancing the generalisability of research findings.^16^ Furthermore, CPRD integrates with other datasets through trusted linkages, including Hospital Episode Statistics Admitted Patient Care data (HES APC) for hospital admissions, mortality records from the Office for National Statistics (ONS) and socioeconomic data. These linkages enable researchers to analyse healthcare pathways across primary and secondary care. Scientific approval for this study was given by the CPRD Independent Scientific Advisory Committee (ISAC) Study reference ID 23_003650. CPRD has received ethical approval from a National Research Ethics Service Committee (NRES) for all purely observational research using anonymised CPRD data. No additional ethical approval was required for this study.

### Study population and case definition

Patients were eligible for inclusion if they were aged 18 years and older at the time of sarcoidosis diagnosis, had contributed data between January 1st 2003 and December 31st 2023 and their record was labelled as acceptable by CPRD quality control. Linked data were available to 31 March 2021.

We identified individuals with sarcoidosis using a comprehensive list of 132 diagnostic codes derived from primary care coding schemes (SNOMED-CT). In individuals with linked data, secondary care codes (International Classification of Diseases, tenth revision [ICD-10]) were used to confirm diagnosis and determine the date of first recorded code (Supplementary 1). Incident diagnoses were defined as the first ever record of sarcoidosis in primary or secondary care records during the study period. The index date was defined as the date of incident sarcoidosis. Individuals who had a diagnosis of sarcoidosis before the study start date or within the first 12 months of registration with their general practice were excluded from incident calculation. Diagnoses made within the first year of registration are more likely to reflect historical conditions, as a patient's full medical history may not yet be transferred or recorded in the CPRD. This exclusion reduces the risk of misclassifying pre-existing (prevalent) cases as incident cases and aligns with well-established methodological practices in CPRD-based research.

### Patient characteristics

For patients with incident sarcoidosis, we extracted data on patient characteristics, including socioeconomic status, ethnicity, smoking status, body mass index (BMI), and comorbidities diagnosed before or at the time of the sarcoidosis diagnosis (sarcoidosis index date). We evaluated eight common comorbidities: diabetes, hypertension, COPD, ischaemic heart disease, chronic kidney disease, congestive cardiac failure, stroke and cancer. For these variables, the most recent code prior to the sarcoidosis index date was recorded. To describe socioeconomic status, we used the Index of Multiple Deprivation (IMD) 2015 quintile. Ethnicity data were extracted from both primary and secondary care records, with the closest code to sarcoidosis index date recorded.

### Matched participants without sarcoidosis

A matched non-sarcoidosis comparator cohort was constructed within the CPRD dataset. Controls were randomly selected from individuals without sarcoidosis for each case based on age (within a 2-year range), sex, and index date for sarcoidosis in a 4:1 ratio from the same primary care practice. Individuals with a prior or new diagnosis of sarcoidosis during follow-up were excluded. This method provided matches for 99% of the sarcoidosis population. The non-sarcoidosis patients were assigned the index date of their matched case.

### Statistical analysis

Characteristics at diagnosis (sarcoidosis index date) were presented for the incident population. For categorical variables, frequencies refer to complete cases. Number and percentage of records with missing data are displayed for variables with missing entries. Incidence was calculated by dividing the number of incident cases by the total person-years in the CPRD. Point prevalence was calculated by dividing the number of people with at least one code for sarcoidosis at mid-calendar year by the number of individuals contributing data to CPRD at the same time point. Sensitivity analyses were performed restricting the prevalent cohort to individuals with i) two or more codes for sarcoidosis issued on two different occasions, ii) a primary care code for sarcoidosis, whether first, second, or subsequent, from 2003 onwards. Age and sex-specific rates were computed. Standardised rates were calculated by applying direct age and sex standardisation to the 2013 European Standard Population.^17^ The European Standard Population is an artificial population structure, designed and published by the statistical office of the European Union, to allow the calculation of age-standardised and sex-standardised rates that are comparable across regions and time. Confidence intervals at the 95% level were calculated using the normal approximation (Wald method). Variance-weighted least-squares regression, incorporating standard errors was used to examine trends in annual incidence over the study period. The normality of residuals was assessed using histograms, while scatter plots of residuals versus fitted values and the Ramsey test were applied to assess linearity. To explore potential non-linear trends, both restricted cubic splines and Joinpoint analysis were employed. To account for heteroscedasticity and autocorrelation, we applied the Newey–West estimator with a two-year, three year and four-year lag. Estimated crude incidence rates for the English population were inferred by applying year-and age-specific incidence to England census mid-year population estimates.

All-cause mortality data were extracted via linkage with ONS national death registries, available for 75% percent of patients. For individuals without data linkage, date of death was extracted from primary care records alone. Annual all-cause mortality rates were calculated for the prevalent population, by dividing the total number of deaths by the number of individuals contributing data to CPRD at the mid-calendar year, and stratified by age, sex and ethnicity. Standardised rates were calculated by applying direct age-and-sex-standardisation to the 2013 European Standard Population. Standardised mortality ratios (SMRs) were calculated by indirect standardisation and derived from the ratio of the number of observed deaths to the number of expected deaths using the general mortality in the population using ONS mortality data from England. Confidence intervals at the 95% level were calculated using the Poisson exact method.

A mixed-effects Poisson regression model with a log link was used to estimate all-cause mortality rates and compare them between the incident sarcoidosis cohort and the matched non-sarcoidosis population. Each control was assigned the index date of their matched case's sarcoidosis diagnosis. The model included a random intercept for each individual to account for within-person correlation, and standard errors were clustered at the matched pair level to account for correlation between cases and their matched controls. Person-years of follow up was accounted for in the analysis. Multivariable adjustment was made for age, gender, ethnicity, year of index date, BMI, smoking status, alcohol intake, and comorbidity selected based on a causal model (Supplementary 2A). A complete case approach was used. Missingness of covariate data was assessed, and sensitivity analyses were conducted to address the impact of missing data (Supplementary 2B). The marginal rates from the regression model were used to estimate adjusted mortality rates per 1000 patient years. Analyses were undertaken using Stata 18.

### Role of the funding source

There was no direct funding source for this research.

## Results

A total of 56,287 patients had a diagnostic code for sarcoidosis prior to December 31st 2023. For incidence calculations, individuals with a code for sarcoidosis before January 1st, 2003 (35,418 records) or within the first 12 months of registration with their general practice (30,858 records) were excluded, leading to 18,554 eligible patients. The mean age of diagnosis in the incident cohort was 51 years (SD 14) and 9883 (53.2%) of these individuals were men. Male patients were diagnosed at a younger age compared with female patients [49 (SD 14) versus 53 (SD 14) p < 0.0001]. The most frequent recorded ethnicity was White (11,333, 63.9%), followed by Mixed (2394, 13.5%), Asian (1724, 9.7%), and Black ethnicities (1448, 8.2%) (Table 1). Compared to the ethnic distribution in England's 2021 Census,^18^ the sarcoidosis cohort had a higher proportion of Black and Mixed ethnicity patients, a lower proportion of White patients, and a similar proportion of Asian individuals (Supplementary 3).

**Table 1:** Characteristics at diagnosis of patients with incident sarcoidosis between 2003 and 2023.

	Total	Gender		IMD quintile		Year of diagnosis	
		Males	Females	Least deprived	Most deprived	2003	2023
	N = 18,564	N = 9888	N = 8674	N = 2962	N = 2743	N = 670	N = 952
Age at diagnosis, years (mean, SD)	50.8 (14.0)	49.2 (13.7)	52.5 (14.1)	52.8 (14.0)	48.5 (13.9)	47.3 (13.4)	53.8 (13.9)
Gender							
Males	9888 (53.3%)	n/a	n/a	1576 (53.2%)	1409 (51.4%)	332 (49.6%)	534 (56.2%)
Females	8674 (46.7%)	n/a	n/a	1386 (46.8%)	1334 (48.6%)	338 (50.4%)	417 (43.8%)
Ethnicity							
White	11,333 (63.9%)	6088 (65.0%)	5245 (62.6%)	2241 (77.8%)	1596 (58.8%)	401 (68.3%)	555 (59.9%)
Black Caribbean or African	1448 (8.2%)	608 (6.5%)	840 (10.0%)	47 (1.6%)	436 (16.1%)	54 (9.2%)	78 (8.4%)
Asian	1724 (9.7%)	898 (9.6%)	826 (9.9%)	185 (6.4%)	373 (13.7%)	44 (7.5%)	91 (9.8%)
Mixed	2394 (13.5%)	1318 (14.1%)	1076 (12.8%)	325 (11.3%)	193 (7.1%)	73 (12.4%)	134 (14.5%)
Other	843 (4.8%)	449 (4.8%)	393 (4.7%)	83 (2.9%)	117 (4.3%)	15 (2.6%)	69 (7.4%)
*Missing ethnicity*	*822 (4.4%)*	*527 (5.3%)*	*294 (3.4%)*	*81 (2.7%)*	*28 (1.0%)*	83 (12.4%)	25 (2.6%)
Socioeconomic status quintile							
Least deprived	2962 (20.6%)	1576 (20.6%)	1386 (20.6%)	n/a	n/a	103 (20.1%)	128 (19.2%)
Most deprived	2743 (19.1%)	1409 (18.4%)	1334 (19.8%)	n/a	n/a	94 (18.3%)	134 (20.1%)
*Missing IMD*	*4175 (22.5%)*	*2225 (22.5%)*	*1949 (22.5%)*	n/a	n/a	*157 (23.4%)*	*284 (29.8%)*
Smoking status							
Non-smoker	9967 (53.7%)	5013 (50.7%)	4954 (57.1%)	1733 (58.5%)	1334 (48.6%)	371 (55.4%)	540 (56.7%)
Ex-smoker	6178 (33.3%)	3449 (34.9%)	2727 (31.4%)	993 (33.5%)	890 (32.4%)	176 (26.3%)	317 (33.3%)
Current smoker	2419 (13.0%)	1426 (14.4%)	993 (11.4%)	236 (8.0%)	519 (18.9%)	123 (18.4%)	95 (10.0%)
Number of comorbidities^a^ (median, IQR)	0.0 (0.0, 1.0)	0.0 (0.0, 1.0)	1.0 (0.0, 1.0)	0.0 (0.0, 1.0)	1.0 (0.0,1.0)	0.0 (0.0, 1.0)	1.0 (0.0, 2.0)
Obesity							
BMI >30 kg/m^2^	6261 (36.4%)	2926 (32.8%)	3334 (40.2%)	837 (30.9%)	1116 (42.8%)	177 (29.4%)	334 (38.8%)
BMI, kg/m^2^ (mean, SD)	28.8 (6.2)	28.4 (5.4)	29.3 (7.0)	28.0 (5.6)	29.7 (6.9)	27.9 (5.9)	29.4 (6.6)
*Missing BMI*	*1346 (7.3%)*	*970 (9.8%)*	*376 (4.3%)*	253 (8.5%)	137 (5.0%)	68 (10.1%)	91 (9.6%)
Alcohol excess or related comorbidity	736 (4.0%)	511 (5.2%)	224 (2.6%)	67 (2.3%)	162 (5.9%)	12 (1.8%)	69 (7.2%)
Diabetes mellitus	2387 (12.9%)	1196 (12.1%)	1189 (13.7%)	285 (9.6%)	474 (17.3%)	56 (8.4%)	174 (18.3%)
Hypertension	4356 (23.5%)	2067 (20.9%)	2289 (26.4%)	643 (21.7%)	707 (25.8%)	120 (17.9%)	263 (27.6%)
COPD	691 (3.7%)	382 (3.9%)	309 (3.6%)	76 (2.6%)	138 (5.0%)	8 (1.2%)	40 (4.2%)
Ischaemic heart disease	1032 (5.6%)	639 (6.5%)	393 (4.5%)	162 (5.5%)	168 (6.1%)	22 (3.3%)	74 (7.8%)
Heart failure	460 (2.5%)	252 (2.5%)	208 (2.4%)	60 (2.0%)	72 (2.6%)	13 (1.9%)	51 (5.4%)
Chronic kidney disease	1036 (5.6%)	455 (4.6%)	581 (6.7%)	154 (5.2%)	165 (6.0%)	9 (1.3%)	67 (7.0%)
Stroke or TIA	460 (2.5%)	252 (2.5%)	208 (2.4%)	69 (2.3%)	66 (2.4%)	14 (2.1%)	41 (4.3%)
Cancer	657 (3.5%)	202 (2.0%)	455 (5.2%)	136 (4.6%)	84 (3.1%)	16 (2.4%)	52 (5.5%)

Abbreviations: BMI, body mass index; IQR, interquartile range; COPD, chronic obstructive pulmonary disease; TIA, transient ischaemic attack.

a Number of comorbidities at sarcoidosis index date = One or more of the following: diabetes, hypertension, chronic obstructive pulmonary disease, ischaemic heart disease, chronic kidney disease, congestive cardiac failure, stroke/transient ischaemic attack and cancer. Number and percentage of records with missing data are displayed for variables with missing entries. For variables with missing entries, category percentages are calculated out of participants with complete data and percentages for missing data are calculated out of total participants.

### Incidence

**Fig. 1:**
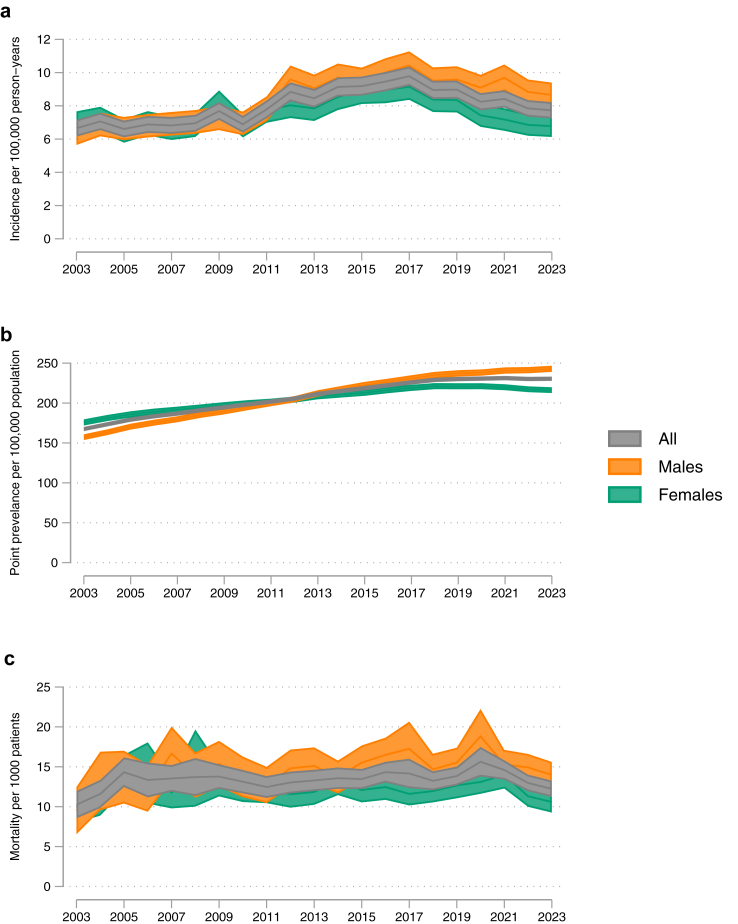
Age and sex standardised incidence, prevalence and mortality of Sarcoidosis. a. Incidence of sarcoidosis per 100,000 person-years b. Point prevalence of sarcoidosis per 100,000 population c. All-cause mortality of sarcoidosis per 1000 population.

The age and sex standardised incidence of sarcoidosis was 6.65 per 100,000 person-years in 2003 and 7.73 per 100,000 person-years in 2023 (Fig. 1a). Regression analyses revealed non-linear trends in annual incidence. Restricted cubic splines identified three distinct phases: a gradual initial increase, a steeper rise, and a decline. Joinpoint analysis confirmed this pattern, detecting breakpoints in 2010 and 2016 and indicating shifts in the rate of incidence change: [estimated annual trend in incidence per 100,000 person-years: pre-2010: 0.10 (95% CI: 0.03–0.17), 2010 to 2016: 0.40 (95% CI: 0.33–0.48) and post 2016: −0.7 (95% CI −0.84 to −0.57)] (Supplementary Figure 4A). When restricting the analysis to pre-COVID pandemic years (2003–2019), a linear trend was observed with an annual increase at 0.20 cases per 100,000 person-years (95% CI 0.18–0.23). There were minimal differences between standardised and crude incidence rates (Supplementary Figure 4B). An estimated absolute number of yearly new diagnoses based on the English census population data increased from 2496 in 2003 to 3393 in 2023 (Supplementary Figure 4C).

The age-and sex-standardised incidence of sarcoidosis was similar between men and women. From 2011 onward, rising incidence rates were primarily driven by an increase in new diagnoses among males, leading to a higher incidence in males compared to females by 2023 [males: 8.65 per 100,000 person-years versus females: 6.78 per 100,000 person-years; IRR 1.28]. Throughout the study period, the crude incidence of sarcoidosis was highest among those aged 40–60 years. However, there was a notable increase in new diagnoses in the over-60-year-old cohort from 2011 onwards. This is reflected in the rising age at presentation [mean age in 2003: 47 years, SD 14; in 2023: 54 years, SD 14] (Table 1 and Supplementary Figure 4D).

### Prevalence

The prevalence of sarcoidosis, identified using at least one diagnostic code and standardised by age and sex, was 167 per 100,000 in 2003 and 230 per 100,000 in 2023 (Fig. 1b). Minimal differences were observed between standardised and crude rates (Supplementary Figure 5A). Prevalence was higher in females until 2013, when the rate in males began to surpass that in females [2023: males: 243 per 100,000 compared to females: 216 per 100,000].

Sensitivity analyses, which restricted the prevalent cohort to individuals with two or more primary care codes for sarcoidosis, showed a lower overall prevalence: 121 per 100,000 in 2023. Similarly, restricting the cohort to individuals with a primary care code for sarcoidosis (whether first, second, or subsequent) from 2003 onwards, yielded a lower overall prevalence: 168 per 100,000 in 2023 (Supplementary Figure 5B).

### Mortality

The age-and-sex-standardised all-cause mortality rate among the prevalent population was 10.2 per 1000 patients in 2003 and 12.2 per 1000 patients in 2023 (Fig. 1c). Regression analyses identified non-linear trends in annual mortality. Restricted cubic splines revealed three distinct phases: a sharp initial increase, followed by a more gradual rise, and a decline after 2021. Joinpoint analysis confirmed this pattern, identifying breakpoints in 2005 and 2021, with estimated annual mortality trends per 1000 patients of 1.43 (95% CI: 0.53–2.32) before 2005, 0.65 (95% CI: −0.01 to 0.14) from 2005 to 2021, and −1.05 (95% CI: −1.68 to −0.41) after 2021 (Supplementary Figure 6A). When restricting the analysis to pre-COVID years (2003–2019), a linear trend was observed, with an annual increase of 0.10 per 1000 patients (95% CI: 0.03–0.18). Standardised mortality rates remained consistently lower than crude rates from 2007 onwards, resulting in a more moderate increase in mortality over the study period (Supplementary Figure 6B). Mortality rates were higher in males compared to females from 2009 until the end of the study period.

**Fig. 2:**
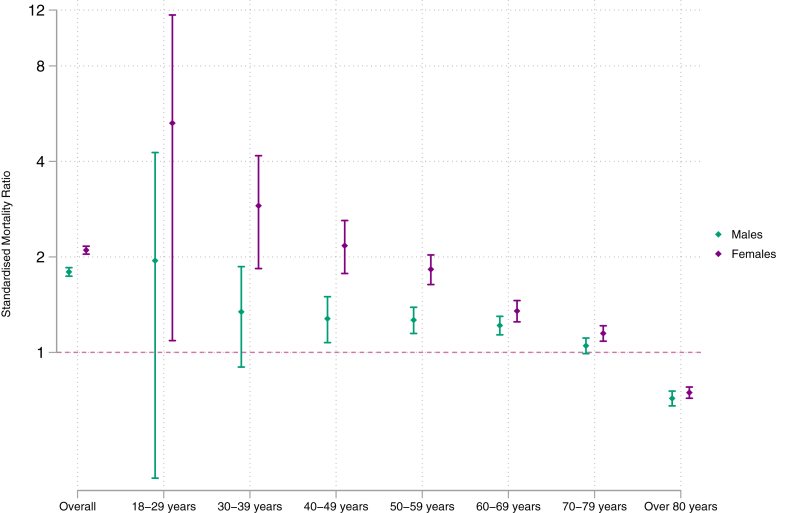
Standardised Mortality Ratios (SMRs) across study duration presented by age and sex. An SMR greater than 1 indicates that all-cause mortality rates in the sarcoidosis cohort is greater than that seen in the general population.

To calculate the standardised mortality ratio (SMR), ONS data on age-specific deaths in England were used (available from 2006 onwards). Relative to the general population, elevated all-cause mortality was observed in both males [SMR: 1.8 (1.7–1.8) and females [SMR: 2.1 (2.0–2.2)] (Fig. 2). SMRs exceeded 1 in each decade of age from 30 to 69, with females consistently showing higher SMRs than males. For individuals over 70, however, the SMR was 1 or below, indicating that all-cause mortality in this age group was similar to or lower than that of the general population. Annual SMRs were elevated in males and females aged 18–49 years old, although confidence intervals were wide, and levels fluctuated during the study period. A more modest elevation was observed in males and females aged 50 to 69 (Supplementary Figure 6C).

Of the 18,556 incident cases diagnosed between 2003 and 2023, 1848 died during follow-up. On average, individuals with sarcoidosis died 2 years younger than their matched comparators (median age at death 72 (62–80) years versus 74 (63–82) p < 0.0001 (Supplementary Table 7A). The age-and sex-adjusted mortality rate was 14.4 per 1000 person-years in incident sarcoidosis cases (95% CI 13.7–15.0) and 10.8 per 1000 person-years in matched comparators (95% CI 10.5–11.0) (Table 2). After adjustment for key covariates, individuals with sarcoidosis had a 34% higher mortality rate compared to matched controls: mortality rate ratio 1.36 (95% CI 1.27–1.44), p < 0.0001. A trend towards increased risk was seen across all age groups from 50 years onwards. Missing data were identified for ethnicity, BMI and IMD. Sensitivity analyses conducted to assess the impact of these missing data, yielded estimates consistent with the primary analysis (Supplementary 7B).

**Table 2:** All-cause mortality: sarcoidosis incident cohort versus matched cohort.

	Sarcoidosis			Matched control population			Mortality rate ratio (95% CI)	
	Deaths	Person-years exposed	Mortality rate per 1000 person-years (95% CI)	Deaths	Person-years exposed	Mortality rate per 1000 person-years (95% CI)	Age and sex adjusted model	Fully adjusted model
Overall	1848	146,357	14.4 (13.7–15.0)	5616	530,601	10.8 (10.5–11.0)	1.34 (1.27–1.41), p < 0.0001	1.36 (1.27–1.44), p < 0.0001
Sex								
Females	915	70,053	14.4 (13.7–15.1)	2709	257,062	10.8 (10.4–11.2)	1.38 (1.28–1.48), p < 0.0001	1.36 (1.24–1.48), p < 0.0001
Males	933	76,304	14.3 (13.6–15.0)	2907	273,539	10.7 (10.4–11.1)	1.30 (1.21–1.40), p < 0.0001	1.35 (1.24–1.48), p < 0.0001
Age at diagnosis								
18–29	5	7863	0.8 (0.8–0.9)	20	25,813	0.6 (0.6–0.7)	0.79 (0.29–2.13), p = 0.64	–
30–39	68	27,987	2.0 (1.9–2.2)	167	93,006	1.5 (1.4–1.6)	1.36 (0.99–1.88), p = 0.059	1.37 (0.93–2.04), p = 0.11
40–49	185	41,292	4.8 (4.5–5.1)	553	143,201	3.6 (3.4–3.8)	1.15 (0.95–1.39), p = 0.14	–
50–59	370	36,882	10.8 (10.3–11.3)	1037	136,045	8.1 (7.9–8.4)	1.34 (1.18–1.53), p < 0.0001	1.43 (1.22–1.68), p < 0.0001
60–69	509	21,122	25.5 (24.4–26.7)	1466	84,106	19.1 (18.6–19.6)	1.42 (1.26–1.59), p < 0.0001	1.49 (1.30–1.71), p < 0.0001
70–80	513	9452	56.2 (53.5–59.0)	1675	40,258	42.1 (40.8–43.3)	1.38 (1.25–1.52), p < 0.0001	1.35 (1.19–1.52), p < 0.0001
>80	198	1758	113.7 (107.3–120.1)	698	8172	85.1 (81.7–88.5)	1.41 (1.20–1.65), p < 0.0001	1.36 (1.13–1.63), p = 0.0013

Mortality rates estimated using Poisson regression models adjusted for age and sex.

Mortality rate ratio estimated using Poisson regression. Model 1 was adjusted for age and sex. Model 2 adjusted for age and sex, ethnicity, year of exposure start, smoking status and comorbidity at index date (obesity, diabetes, hypertension, chronic obstructive pulmonary disease, ischaemic heart disease, chronic kidney disease, congestive cardiac failure, stroke/transient ischaemic attack and cancer). The model did not converge for age groups 18–29 and 40–49 in the fully adjusted model.

## Discussion

This population-based study represents the most comprehensive examination of sarcoidosis incidence in England, utilising the largest cohort to date and surpassing previous data that are over 20 years old. This is also the first study to describe the prevalence of sarcoidosis in England. Both incidence and prevalence have increased during the study period with notable shifts in age and sex distribution. There has been a small increase in all-cause mortality, with rates that exceed those of the general population, particularly amongst individuals aged 30–70 years. This higher risk of death was also observed in the incident cohort when compared to an age-and-sex-matched population within the CPRD.

Our incidence estimate of sarcoidosis is higher than previous estimates. While the overall incidence increased during the study period, the rate in the first year of our study, 2003, was only marginally higher than the rate reported for the 2000–2003 period in previously published data.^12^ In comparison to global rates, the incidence in the UK is now comparable to that of Canada^4^ and the USA,^8^ but remains lower than Sweden^3^ and Denmark.^19^ We estimated the prevalence of sarcoidosis at 230 per 100,000 people in 2023. However, sensitivity analyses using stricter criteria yielded lower estimates (121 per 100,000), suggesting the true prevalence lies between these values. Our upper-bound estimate of prevalence in England is higher than those reported in the USA^8^ and Denmark,^19^ but aligns with calculations from Switzerland,^20^ Sweden,^3^ and Canada.^4^ The change in prevalence during the study period may reflect the longer observation period of data. As seen in other chronic conditions, extended observation periods allow for the cumulative inclusion of cases over time, and if there are favourable survival rates, the number of individuals living with the condition continues to rise.^21^

It is widely acknowledged that the incidence and prevalence of sarcoidosis varies across the world due to a combination of genetic, environmental, and socio-demographic factors. However differences in estimates may also relate to the choice of dataset and the definition of sarcoidosis which can lead to over- or underestimation.^22^ We defined sarcoidosis by primary care codes, which captures a broader range of cases, including milder or early-stage disease, but may be less precise. In contrast, epidemiological studies using hospital admissions or outpatient data may be more accurate, reflecting severe or complex cases, but may miss those diagnosed or managed solely in primary care.^23^ Differences in age and sex distribution between countries can also complicate comparisons of incidence and prevalence.^22^ In this study, we standardised our rates by age and sex to enable more accurate cross-population comparisons. However, not all studies apply this standardisation, which makes direct comparisons challenging.

We observed that sarcoidosis incidence did not increase at a constant rate but followed a complex trajectory with periods of acceleration and decline. The most pronounced rise occurred between 2010 and 2016. This trend aligns with findings from other longitudinal studies.^19^^,^^24^ In Denmark, the sharpest increase was reported in individuals who were not receiving treatment for their sarcoidosis, with the authors suggesting that growing use of diagnostic imaging may have led to more incidental findings of sarcoidosis.^19^ Similarly, in England, diagnostic techniques, such as the increased availability of FDG PET-CT scans and endobronchial ultrasound,^25^ have likely contributed to the rise in new diagnoses. The publication of key guidelines during this period, including the WASOG Criteria for Organ Involvement in Sarcoidosis^26^ and the Heart Rhythm Society Consensus Statement on Cardiac Sarcoidosis^27^ may also have played a significant role. These publications likely improved awareness among healthcare providers, standardised diagnostic practices, and facilitated earlier detection of the disease. Other potential explanations for the rising incidence include increasing obesity^28^, ^29^, ^30^ and possible, yet unknown, changes in environmental or occupational exposures.^20^

Although the primary objective of our study was not to investigate the impact of the COVID-19 pandemic, analysis restricted to the pre-pandemic years (2003–2019) demonstrated a numerically higher annual increase in sarcoidosis incidence compared to analyses that included the pandemic years. While this specific trend has not been reported for sarcoidosis, the pandemic has been linked to a significant reduction in the diagnosis of other conditions.^31^^,^^32^ It is possible that the pandemic had a differential impact on sarcoidosis diagnoses across age groups, potentially influencing the observed differences in incidence trends, although we lack evidence to confirm this.

We observed shifts in age and sex distribution, with a rising incidence among males and those over 60 years. Similar trends have been noted in other countries.^3^^,^^4^^,^^8^^,^^19^^,^^33^^,^^34^ Additionally, we found that males had a younger age of onset compared to females, a pattern now recognised.^3^^,^^35^^,^^36^ The observed second peak in disease onset may reflect increased incidental diagnoses in a highly medicalised population with age-related comorbidities. Alternatively, it could represent a distinct pathophysiological subset of sarcoidosis in older adults, potentially driven by immunosenescence or prolonged exposure to triggers such as infections or organic antigens.^37^ In females, hormonal changes after menopause may modulate immune responses with loss of the potential protective effect of oestrogen^35^

Although mortality estimates for sarcoidosis vary across studies due to differences in demographics, disease severity, and healthcare settings, an increasing risk of death overtime has been consistently observed.^4^^,^^9^ In our study, the observed rise in all-cause mortality attenuated after age and sex standardisation, suggesting that changes in population structure contributed to the increase in crude mortality rates. Several studies have compared the mortality rate in sarcoidosis with that of the general population.^38^ In the Black Women's Health Study, women with sarcoidosis were more than twice as likely to die as women without the disease, which was persistent across all age groups.^39^ After adjusting for key covariates, we demonstrated that individuals with sarcoidosis had a 36% higher mortality rate compared to matched controls (mortality rate ratio 1.36 (95% CI 1.27–1.44), a risk that remained consistent across age groups from 50 years onwards. The narrow confidence interval suggests a precise estimate; however, its validity depends on the absence of unmeasured confounding, which should be considered when interpreting these findings.

A major strength of our study is the validated population-level data source used, containing pseudonymised data on over 55,000 patients with sarcoidosis, and covering a period of 20 years.^40^ Data is representative of the broader English population in terms of geographical spread, deprivation as well as age and gender.^41^

There are several limitations of this study. Firstly, the reliance on primary care data to identify sarcoidosis codes, despite sarcoidosis typically being diagnosed in secondary care, except for classical Löfgren's syndrome. The UK does not have a centralised electronic health record (EHR) system that integrates data across all secondary care outpatient settings. Consequently, information generated during outpatient consultations remains difficult to access for research purposes. It is however standard practice in the UK for new diagnoses made in secondary care to be recorded in primary care EHRs. Validation studies have evaluated the completeness of GP-recorded diagnoses originating from hospital consultations, with high accuracy and completeness.^42^ As CPRD does not capture granular data from routine outpatient hospital care, we did not have information on the specialty responsible for the diagnosis of sarcoidosis, whether histological confirmation was obtained, imaging results, and medications prescribed exclusively in outpatient settings. While prescriptions for corticosteroids and steroid-sparing medications from primary care were reviewed, these data are not presented as they likely underestimate the actual treatment rates within the cohort and may introduce bias. The limited availability of detailed data on sarcoidosis phenotypes posed a challenge in identifying the number of individuals with Löfgren's syndrome, a distinct subset of the disease. This phenotype was rarely coded in the dataset, yet its separate reporting on incidence and prevalence could have offered valuable insights.

Second is the potential for diagnostic misclassification, as it depends on the precision and reliability of clinicians or administrative staff tasked with entering the codes. The validity of a sarcoidosis code in the CPRD has not been examined, though other disease codes show high positive predictive values.^40^ Validation studies of secondary care codes for sarcoidosis indicate that using two or more codes improves confirmation rates.^43^ Applying a similar requirement in a primary care setting may lead to under-reporting as most patients with sarcoidosis are managed in secondary care and may only receive a primary care code if presenting to their general practitioner with an acute issue relating to sarcoidosis.

Thirdly, we did not have denominator data broken down by ethnic group, which limited our ability to present incidence and prevalence by ethnicity. This could have provided valuable insights into potential disparities in disease patterns and outcomes across different ethnic groups.

Fourthly, mortality data were obtained from linked ONS death registrations, which is the gold standard for mortality research in England and Wales due to its high coverage and reliability. However, linkage to ONS data was available for only 75% of the study cohort, and mortality data from 2021 onwards are not yet available. CPRD also captures deaths, and while the date and cause of death may be less reliable compared to ONS data, the overall identification of deaths closely aligns with ONS records^44^ We acknowledge that these linkage limitations may have led to an underestimation of mortality and could have influenced the analysis of time trends. Reassuringly sensitivity analyses of mortality rate ratios, restricted to cases and matched controls with linked data, yielded results consistent with the primary analysis.

Lastly, there is the potential impact of unmeasured confounders, such as lifestyle factors, or residual confounding arising from measurement errors in variables like smoking status. These factors may have influenced the effect estimates from our models examining mortality risk, highlighting the challenges of accurately capturing complex behaviours in observational studies.

In conclusion, the incidence of sarcoidosis in England is rising, with notable shifts in age and sex distribution. Mortality rates have increased, with younger patients facing a higher risk of death compared to the general population. Recognising the burden of disease and its associated mortality is crucial for refining healthcare policies, optimising resource allocation and ultimately improving patient outcomes.

## Contributors

Conceptualisation: KB and JBG. Methodology: KB, JBG, SN, MDR, SP. Data curation: JBG, KB, AD. Formal analysis: KB, JBG, and SN. Interpretation of findings: all authors. Writing of the original draft: KB. Revising, review, and editing: all authors. All authors read and approved the final manuscript. KB and JBG are the guarantors for the article and accept full responsibility for the work and the conduct of the study and controlled the decision to publish. KB and JBG directly accessed and verified the underlying data reported in the manuscript. All authors had full access to all the data in the study, and they accept responsibility for the decision to submit for publication. The corresponding author attests that all listed authors meet authorship criteria and that no others meeting the criteria have been omitted.

## Data sharing statement

The anonymised, coded data used in these analyses were provided by CPRD following approval by their Research Data Governance committee. The data are available on request from CPRD. Additional code lists used in these analyses are available on request from the corresponding author.

## Declaration of interests

KB has received grant funding from the Pfizer/Versus Arthritis; honoraria from Galapagos, UCB, and Viforpharma; and educational support from UCB. JBG has received honoraria from AbbVie, Biovitrum, BMS, Celgene, Chugai, Galapagos, Gilead, Janssen, Lilly, Novartis, Pfizer, Roche, Sanofi, Sobi, and UCB; and grant funding from Sandoz UK. MDR has received honoraria from AbbVie, Lilly, Galapagos, Menarini, UCB, and Viforpharma; grant funding from Sandoz UK; advisory board fees from Biogen; and support for attending educational meetings from Lilly, Pfizer, Janssen, and UCB. SSP have received consulting fees from Atyr and Kinevant. All other authors declare no declaration of interests.
