# Supplementary material for: Incidence, prevalence, and mortality of sarcoidosis in England: a population-based study
Source: Lancet Reg Health Eur. 2025 Apr 4;53:101283. doi: 10.1016/j.lanepe.2025.101283 (PMC12002749; doi:10.1016/j.lanepe.2025.101283)
Supplement: Multimedia component 1 [file mmc1.docx]

**Supplementary 1. Sarcoidosis Codes**

| **SNOMED Code** |  | |
| --- | --- | --- |
| 312642016 | [X]Myositis in sarcoidosis classified elsewhere | |
| 293742013 | [X]Sarcoidosis of other and combined sites | |
| 5.06587E+15 | Acute sarcoid polymyositis | |
| 5.12493E+15 | Acute skin sarcoidosis | |
| 3.98287E+15 | Angiolupoid sarcoidosis | |
| 6.22063E+15 | Arthritis secondary to sarcoidosis | |
| 6.66432E+15 | Atrophic sarcoidosis | |
| 5.0555E+15 | BHL sarcoidosis - Bilateral hilar lymph node sarcoidosis | |
| 5.05551E+15 | Bilateral hilar lymph node sarcoidosis | |
| 5.12497E+15 | Bilateral hilar lymph node sarcoidosis with erythema nodosum | |
| 3.00811E+15 | Boeck's sarcoid | |
| 3.00817E+15 | Boeck's sarcoidosis | |
| 3.72619E+15 | Cardiac sarcoidosis | |
| 4.20139E+15 | Cerebral sarcoidosis | |
| 5.06588E+15 | Chronic sarcoid myopathy | |
| 5.12502E+15 | Chronic skin sarcoidosis | |
| 3.40891E+15 | Cutaneous sarcoidosis | |
| 3.00816E+15 | Darier-Roussy sarcoid | |
| 7.86118E+15 | Demyelination of central nervous system co-occurrent and due to neurosarcoidosis | |
| 7.86119E+15 | Demyelination of central nervous system with neurosarcoidosis | |
| 3514527012 | Digestive sarcoidosis | |
| 2.59568E+15 | Dilated cardiomyopathy secondary to sarcoidosis | |
| 7.87685E+15 | Early onset sarcoidosis | |
| 5.05558E+15 | Endobronchial sarcoidosis | |
| 5.12498E+15 | Erythema nodosum with bilateral hilar lymph node sarcoidosis | |
| 5.12501E+15 | Erythematous sarcoidosis | |
| 3.09703E+15 | Granulomatous sarcoid nephropathy | |
| 2.56915E+15 | Heerfordt syndrome | |
| 2.56913E+15 | Heerfordt's syndrome | |
| 2.56916E+15 | Heerfordt-WaldenstrÃ¶m syndrome | |
| 2.56917E+15 | Heerfordt-Waldenstrom syndrome | |
| 303525019 | Hepatic granulomas in sarcoidosis | |
| 5.05518E+15 | Hilar lymph node sarcoidosis | |
| 2.61297E+15 | Hypercalcaemia due to sarcoidosis | |
| 2.61298E+15 | Hypercalcemia due to sarcoidosis | |
| 6.66437E+15 | Hypomelanosis in sarcoidosis | |
| 6.66436E+15 | Hypomelanotic sarcoidosis | |
| 3.77813E+15 | Hypothyroidism due to sarcoidosis | |
| 6.66431E+15 | Ichthyosiform sarcoidosis | |
| 5.06585E+15 | Lacrimal and parotid gland sarcoidosis | |
| 5.0368E+15 | Laryngeal sarcoidosis | |
| 5.12505E+15 | Lichenoid sarcoidosis | |
| 5.12495E+15 | Lofgrens syndrome | |
| 5.12496E+15 | Lofgren's syndrome | |
| 3.55322E+15 | Lymph node sarcoidosis | |
| 5.125E+15 | Maculopapular sarcoidosis | |
| 5.05517E+15 | Mediastinal lymph node sarcoidosis | |
| 4.7679E+15 | Meningeal sarcoidosis | |
| 296819018 | Meningitis due to sarcoidosis | |
| 297440017 | Multiple cranial nerve palsies in sarcoidosis | |
| 297643013 | Myopathy due to sarcoidosis | |
| 311616019 | Myositis in sarcoidosis | |
| 6.66438E+15 | Nail dystrophy due to sarcoidosis | |
| 5.03537E+15 | Nasal sarcoidosis | |
| 5.06586E+15 | Nasopharyngeal sarcoidosis | |
| 4.19356E+15 | Necrotising sarcoid granulomatosis | |
| 4.19357E+15 | Necrotizing sarcoid granulomatosis | |
| 5.00475E+15 | Neurosarcoidosis | |
| 4.20141E+15 | Nodular sarcoidosis | |
| 5.06584E+15 | Ocular sarcoidosis | |
| 5.06589E+15 | Orofacial sarcoid | |
| 5.12504E+15 | Papular sarcoidosis | |
| 297566017 | Polyneuropathy in sarcoidosis | |
| 5.05557E+15 | Pulmonary fibrosis due to sarcoidosis | |
| 7.50194E+15 | Pulmonary hypertension in sarcoidosis | |
| 5.05555E+15 | Pulmonary sarcoid infiltration | |
| 5.05553E+15 | Pulmonary sarcoid infiltration with bilateral hilar lymphadenopathy | |
| 40879015 | Pulmonary sarcoidosis | |
| 3.09704E+15 | Renal sarcoidosis | |
| 3.12844E+15 | Restrictive cardiomyopathy secondary to sarcoidosis | |
| 6.22062E+15 | Sarcoid arthritis | |
| 477671011 | Sarcoid arthropathy | |
| 6.02324E+15 | Sarcoid chorioretinitis | |
| 5.06582E+15 | Sarcoid dactylitis | |
| 1.61591E+14 | Sarcoid heart disease | |
| 4.77724E+15 | Sarcoid heart muscle disease | |
| 8.11326E+15 | Sarcoid iridocyclitis | |
| 4.76788E+15 | Sarcoid meningitis | |
| 1494958019 | Sarcoid myocarditis | |
| 4.77093E+15 | Sarcoid myopathy | |
| 4.77062E+15 | Sarcoid neuropathy | |
| 5.05559E+15 | Sarcoid pulmonary calcification | |
| 5.02759E+15 | Sarcoid skin of eyelid | |
| 3.57565E+15 | Sarcoid type granuloma | |
| 6.87411E+15 | Sarcoid uveitis | |
| 8.36584E+15 | Sarcoid vasculitis | |
| 6.63515E+15 | Sarcoidal granuloma of skin | |
| 52728016 | Sarcoidosis | |
| 5.12503E+15 | Sarcoidosis in scar | |
| 3514526015 | Sarcoidosis of digestive system | |
| 454209012 | Sarcoidosis of inferior turbinates | |
| 1223250017 | Sarcoidosis of lung | |
| 287930013 | Sarcoidosis of lung with sarcoidosis of lymph nodes | |
| 1232607019 | Sarcoidosis of lymph nodes | |
| 7.6142E+15 | Sarcoidosis of oral cavity | |
| 1231546012 | Sarcoidosis of skin | |
| 7.04349E+15 | Sarcoidosis with glomerulonephritis | |
| 3.98286E+15 | Sarcoidosis, angiolupoid type | |
| 3.45652E+15 | Sarcoidosis, anular type | |
| 2.84666E+15 | Sarcoidosis, Darier-Roussy type | |
| 2.65194E+15 | Sarcoidosis, erythrodermic type | |
| 3.67839E+15 | Sarcoidosis, lupus pernio type | |
| 4.2014E+15 | Sarcoidosis, nodular type | |
| 3.38455E+15 | Sarcoidosis, plaque type | |
| 5.12494E+15 | Sarcoidosis-induced erythema nodosum | |
| 3.40893E+15 | Skin sarcoidosis | |
| 2.77552E+15 | Splenic sarcoidosis | |
| 5.05549E+15 | Stage 1 pulmonary sarcoidosis | |
| 5.05552E+15 | Stage 2 pulmonary sarcoidosis | |
| 5.05554E+15 | Stage 3 pulmonary sarcoidosis | |
| 5.05556E+15 | Stage 4 pulmonary sarcoidosis | |
| 6.66435E+15 | Subcutaneous nodular sarcoidosis | |
| 3.81649E+15 | Subcutaneous sarcoidosis | |
| 6.66433E+15 | Ulcerative sarcoidosis | |
| 6.66434E+15 | Verrucous sarcoidosis | |
| **ICD-10 Codes** |  | |
| D86 | Sarcoidosis |  |
| D86.0 | Sarcoidosis of lung |  |
| D86.1 | Sarcoidosis of lymph nodes |  |
| D86.2 | Sarcoidosis of lung with sarcoidosis of lymph nodes |  |
| D86.3 | Sarcoidosis of skin |  |
| D86.8 | Sarcoidosis of other sites |  |
| D86.81 | Sarcoid meningitis |  |
| D86.82 | Multiple cranial nerve palsies in sarcoidosis |  |
| D86.83 | Sarcoid iridocyclitis |  |
| D86.84 | Sarcoid pyelonephritis |  |
| D86.85 | Sarcoid myocarditis |  |
| D86.86 | Sarcoid arthropathy |  |
| D86.87 | Sarcoid myositis |  |
| D86.89 | Sarcoidosis of other sites |  |
| D86.9 | Sarcoidosis, unspecified |  |
| G53.2 | Multiple cranial nerve palsies in sarcoidosis |  |
| M63.3 | Myositis in sarcoidosis |  |

**Supplementary 2A.** Directed acyclic graph examining the risk of death in people with sarcoidosis compared to the matched controls.

We identified a minimally sufficient set of confounders using a causal framework, constructing a directed acyclic graph (DAG) to assess the risk of death in individuals with sarcoidosis compared to matched controls. The DAG was informed by existing literature and domain expertise to identify potential confounding pathways that could bias the association of interest. We included all covariates that indicated biasing pathways (i.e., confounders) in our models to ensure appropriate adjustment.

The conceptual framework illustrates the assumed relationships between covariates, the primary exposure (sarcoidosis), and the outcome (death). Pink boxes denote variables that are ancestors of both the exposure and the outcome, while blue boxes represent ancestors of the outcome only. Pink lines indicate biasing pathways (i.e., confounding), whereas green lines represent causal pathways.


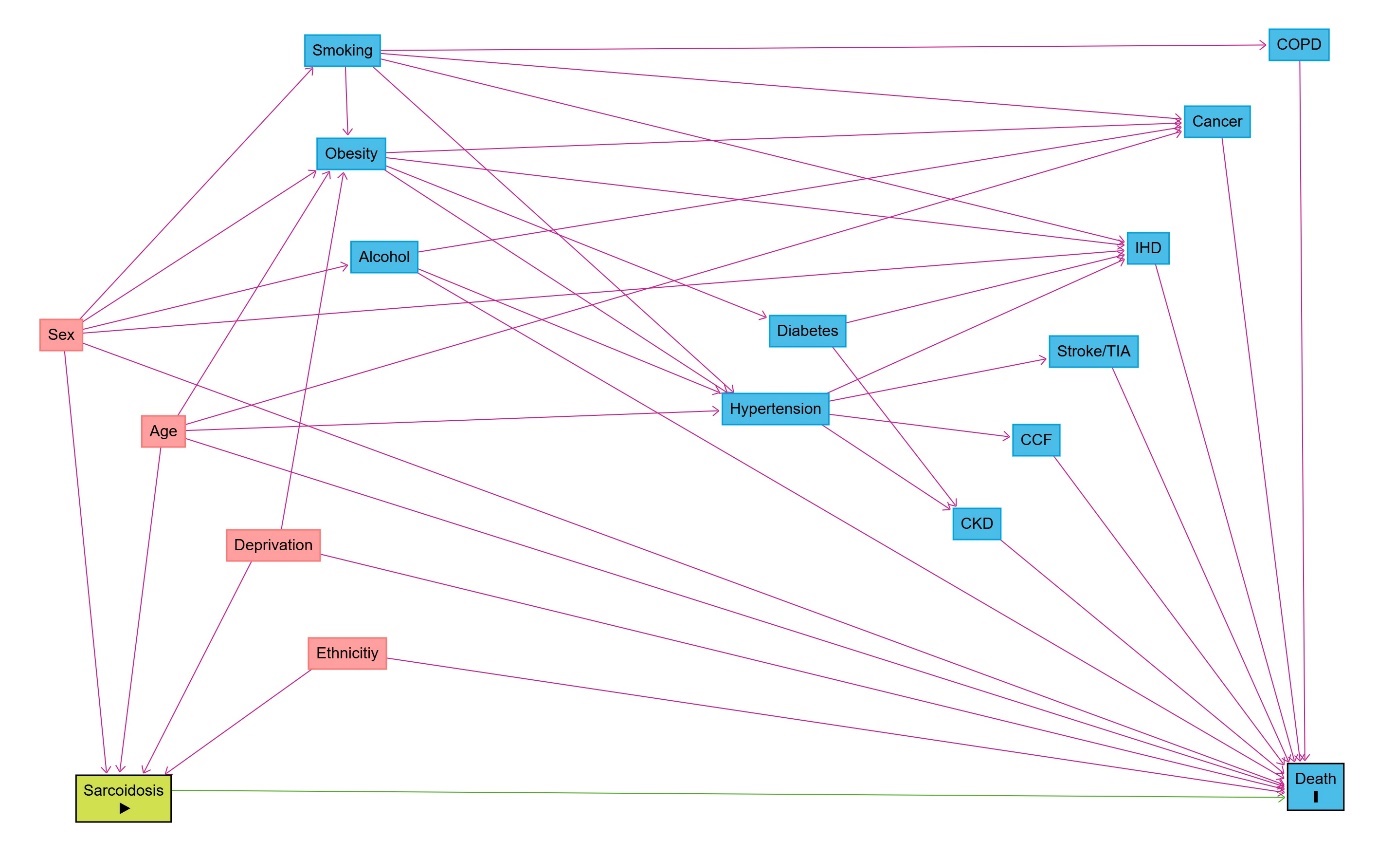


**Supplementary 2B.** Missing data.

Missing data on age and gender were complete for all participants. Smoking status was classified as current, former, or non-smoker, based on codes closest to the sarcoidosis index date. Individuals without recorded smoking status in the CPRD were assumed to be non-smokers. Data on alcohol excess/related comorbidities and specific comorbidities (diabetes mellitus, hypertension, COPD, ischaemic heart disease, heart failure, chronic kidney disease, stroke or TIA, and cancer) were recorded. If corresponding codes were absent in the CPRD observation files, this was assumed to indicate a negative status.

Overall, 35% of the cohort had at least one missing covariate in the regression model. Missing data were primarily observed in BMI, ethnicity, and IMD. The most significant contributor to missing data was IMD, which was unavailable for individuals without linkage.

- Ethnicity data were missing for 4.4% of individuals in the case group and 9.5% in the control group. This data is likely to be missing at random and may be influenced by the context in which healthcare was provided. For instance, patients with poorer health may have been more likely to have their ethnicity recorded due to more frequent interactions with healthcare services compared to healthier individuals (1). The primary analysis took a complete case approach. Sensitivity analyses were conducted categorising missing ethnicity as a separate category "Unknown" in the analysis to maintain a comprehensive dataset,
- Obesity was assessed using BMI values recorded prior to sarcoidosis index date or within 5–10 years of sarcoidosis index date. BMI data were missing for 7.3% of cases and 14.9% of controls. The missing BMI data were likely *missing not at random* (MNAR), meaning the likelihood of missingness was related to the variable itself. For instance, BMI may be more often recorded for obese individuals and likely to be absent for individuals who do not appear overweight. MNAR data cannot reliably be estimated from other variables unless the mechanism driving the missingness is fully understood. Although multiple imputation provides some robustness against MNAR mechanisms, accurately addressing them is challenging. The primary analysis took a complete case approach. To account for missing BMI data, sensitivity analyses were performed assuming all individuals with missing BMI data were non-obese (BMI <30).
- The Index of Multiple Deprivation (IMD) was only available for individuals with linked data. Linked data were unavailable for 4172 (22.5%) cases and 17,191 (23.2%) controls. Among those with linkage, IMD data were missing for 34 (0.2%) cases and 160 (0.3%) controls. To address the issue of missing IMD data, a sensitivity analysis was performed restricting the analysis to cases and matched controls with linked data. This approach not only reduced the proportion of missing IMD data but also enabled an analysis restricted to individuals with linked ONS mortality data.

Table. Missing Data for Case and Controls included the analyses.

| Variable | Cases: Missing Variables (n, %) | | Controls: Missing Variables (n, %) | |
| --- | --- | --- | --- | --- |
| Age at diagnosis | 0 of 18560 | 0% | 0 of 74215 | 0% |
| Gender | 0 of 18560 | 0% | 0 of 74215 | 0% |
| Ethnicity | 821 of 18560 | 4.4% | 7057 of 74215 | 9.5% |
| Smoking status | 0 of 18560 | 0% | 0 of 74215 | 0% |
| BMI | 1346 of 18560 | 7.3% | 11,094 of 74215 | 14.9% |
| IMD: all population  linked population | 4172 of 18560  34 of 14422 | 22.5%  0.2% | 17191 of 74215  160 of 57184 | 23.2%  0.3% |
| Alcohol excess or related comorbidity | 0 of 18560 | 0% | 0 of 74215 | 0% |
| \| Diabetes mellitus \| \| --- \| | 0 of 18560 | 0% | 0 of 74215 | 0% |
| \| Hypertension \| \| --- \| | 0 of 18560 | 0% | 0 of 74215 | 0% |
| \| COPD \| \| --- \| | 0 of 18560 | 0% | 0 of 74215 | 0% |
| \| Ischaemic heart disease \| \| --- \| | 0 of 18560 | 0% | 0 of 74215 | 0% |
| \| Heart failure \| \| --- \| | 0 of 18560 | 0% | 0 of 74215 | 0% |
| \| Chronic kidney disease \| \| --- \| | 0 of 18560 | 0% | 0 of 74215 | 0% |
| \| Stroke or TIA \| \| --- \| | 0 of 18560 | 0% | 0 of 74215 | 0% |
| Cancer | 0 of 18560 | 0% | 0 of 74215 | 0% |

**Reference:**

1. Shiekh SI, Harley M, Ghosh RE, Ashworth M, Myles P, Booth HP, et al. Completeness, agreement, and representativeness of ethnicity recording in the United Kingdom’s Clinical Practice Research Datalink (CPRD) and linked Hospital Episode Statistics (HES). Population Health Metrics. 2023;21(1):3.

**Supplementary table 3**. Comparison of ethnicity in the incident sarcoidosis cohort compared to the ethnicity reported in England 2021 Census data, presented as a ratio.

|  | Incident cohort (count) | Incident cohort (%) | Census 2021 (%) | Ratio incident cohort: census |
| --- | --- | --- | --- | --- |
| Ethnicity |  |  |  |  |
| White | 11333 | 63.9 | 81.7 | 0.78 |
| Black Caribbean or African | 1448 | 8.2 | 4.0 | 2.05 |
| Asian | 1724 | 9.7 | 9.3 | 1.04 |
| Mixed | 2394 | 13.5 | 2.9 | 4.66 |
| Other | 843 | 4.8 | 2.1 | 2.29 |

To understand the ethnicity of individuals with sarcoidosis, it is essential to consider the ethnic composition of the denominator population. However, we did not have access to CPRD denominator data stratified by ethnicity. Instead, we compared the ethnic composition of the incident sarcoidosis cohort with the most recent data on England's population from the 2021 Census. Our analysis revealed that the proportion of Black patients with sarcoidosis is nearly double that of Black individuals in the general population, while the proportion of patients of Mixed ethnicity with sarcoidosis is more than four times higher than Mixed ethnicity individuals in the general population. In contrast, the proportion of White patients with sarcoidosis is lower than in the general population of England, and there is no observed difference in the proportion of Asian individuals.

**Supplementary figure 4A**. Analysis of non-linear trends in incidence.

Regression analysis using a variance-weighted least squares regression line showed that the incidence of sarcoidosis increased by 0.10 cases per 100,000 person-years annually (95% Confidence Interval: 0.09 to 0.12) over the study period. However, when restricted cubic splines were used to explore non-linear trends in incidence, three distinct phases emerged: a slower initial increase in incidence, a more rapid rise in cases after the initial phase and a decline. Joinpoint analysis, which identifies points where trends change significantly, confirmed this pattern, detecting breakpoints in 2010 and 2016 indicating shifts in the rate of incidence change: [estimated annual trend in incidence per 100,000 person-years: pre 2010: 0.10 (95% CI: 0.03 to 0.17), 2010 to 2016: 0.40 (95% CI: 0.33 to 0.48) and post 2016: - 0.7 (95% CI -0.84 to -0.57)]. To account for heteroscedasticity and autocorrelation, we applied the Newey-West estimator with a two, three and four-year lag, ensuring robust standard errors. The model successfully converged and produced the same breakpoints as our initial analysis, with similar estimated rates of change.

This figure visually presents these findings. Both restricted cubic spline analysis and joinpoint analysis support the conclusion that sarcoidosis incidence did not increase at a constant rate but instead followed a more complex trajectory with periods of acceleration and decline.

**Supplement Figure 4B.** Crude versus age and sex- standardised incidence of sarcoidosis.

**Supplement Figure 4C**. Estimated absolute numbers of new diagnosis based on England census population.


**Supplement Figure** 4**D**. Incidence of sarcoidosis by age group.

**Supplementary figure 5A**. Crude versus age and sex- standardised point prevalence of sarcoidosis.

**Supplementary figure 5B**. Sensitivity analyses age and sex- standardised point prevalence of sarcoidosis, presented overall and by gender restricting the prevalent cohort to individuals with i) two or more codes for sarcoidosis issued on two different occasions, ii) a primary care code for sarcoidosis, whether first, second, or subsequent, from 2003 onwards.


**Supplementary figure 6A.** Analysis of non-linear trends in mortality.

Regression analysis using a variance-weighted least squares regression line showed that there was an annual increase in mortality of 0.05 per 1,000 patients (95% CI -0.01 to 0.10), Further analysis confirmed that this increase did not follow a simple linear trend. Restricted cubic splines revealed distinct phases: a step initial increase, followed by a more gradual rise, and then a decline after 2021. Joinpoint analysis, which identifies points where trends change significantly, confirmed this pattern, detecting breakpoints in 2005 and 2021 [estimated annual trend in incidence per 100,000 person-years: pre-2005: 1.43 (95% CI: 0.53 to 2.32), post 2005 to 2021: 0.65 (95% CI: -0.01 to 0.14) and post 2021: - 1.05 (95% CI -1.68 to -0.41)]. To account for heteroscedasticity and autocorrelation, we applied the Newey-West estimator with a two, three and four-year lag, ensuring robust standard errors. The model successfully converged and produced the same breakpoints as our initial analysis, with similar estimated rates of change.

**Supplementary figure 6B.** Age and sex- standardised mortality in sarcoidosis


**Supplementary figure 6C.** SMRs by individual year, present by gender and age group

**Supplementary table 7A**. Table comparing baseline data between cases and matched controls

|  | Incident Sarcoidosis Population | Matched Control Population |
| --- | --- | --- |
|  | N=18,560 | N=74,215 |
| Ethnicity |  |  |
| White | 11,333 (63.9%) | 55,769 (83.0%) |
| Black Caribbean or African | 1,448 (8.2%) | 3,304 (4.9%) |
| Asian | 1,723 (9.7%) | 6,036 (9.0%) |
| Mixed | 2,394 (13.5%) | 684 (1.0%) |
| Other | 841 (4.7%) | 1,365 (2.0%) |
| *Missing ethnicity* | 821 (4.4%) | 7,057 (9.5%) |
| Socioeconomic status quintile |  |  |
| Least deprived | 2,962 (20.6%) | 11,447 (20.1%) |
| Most deprived | 2,743 (19.1%) | 10,770 (18.9%) |
| *Missing IMD* | 4,172 (22.5%) | 17,191 (23.2%) |
| Smoking status |  |  |
| Non-Smoker | 9,967 (53.7%) | 36,714 (49.5%) |
| Ex-smoker | 6,175 (33.3%) | 21,279 (28.7%) |
| Current smoker | 2,418 (13.0%) | 16,222 (21.9%) |
| Obesity |  |  |
| BMI >30 kg/m^2^ | 6,259 (36.4%) | 17,536 (27.8%) |
| BMI, kg/m^2^ (mean, SD) | 28.8 (6.2) | 27.6 (5.8) |
| *Missing BMI* | 1,346 (7.3%) | 11,094 (14.9%) |
| Number of comorbidities at baseline | 0.0 (0.0,1.0) | 0.0 (0.0,1.0) |
| Alcohol excess or related comorbidity | 735 (4.0%) | 3,233 (4.4%) |
| Diabetes | 2,385 (12.9%) | 5,530 (7.5%) |
| Hypertension | 4,356 (23.5%) | 13,938 (18.8%) |
| Asthma | 3,326 (17.9%) | 9,949 (13.4%) |
| COPD | 691 (3.7%) | 2,051 (2.8%) |
| Ischaemic heart disease | 1,032 (5.6%) | 3,278 (4.4%) |
| Heart failure | 460 (2.5%) | 798 (1.1%) |
| Chronic kidney disease | 1,036 (5.6%) | 2,193 (3.0%) |
| Stroke or TIA | 460 (2.5%) | 1,472 (2.0%) |
| Cancer | 657 (3.5%) | 1,567 (2.1%) |
| Linked data to ONS | 14,422 (77.7%) | 57,184 (77.1%) |

Abbreviations: BMI = body mass index. interquartile range; COPD = chronic obstructive pulmonary disease, TIA: transient ischaemic attack. Number of comorbidities at sarcoidosis index date = One or more of the following: , diabetes, hypertension, chronic obstructive pulmonary disease, ischaemic heart disease, chronic kidney disease, congestive cardiac failure, stroke/transient ischaemic attack and cancer. Number and percentage of records with missing data are displayed for variables with missing entries. For variables with missing entries, category percentages are calculated out of participants with complete data and percentages for missing data are calculated out of total participants.

In total, 4 patients with incident sarcoidosis did not have a matched control. Nearly all other individuals (n=18,544) had 4 matched controls; 14 individuals had 3 matched controls, and 2 individuals had 2 matched controls.

**Supplementary 7B.** Sensitivity analyses

Sensitivity analyses were conducted to address missing ethnicity, BMI and IMD data. These models were adjusted as per the primary analysis, whilst also:

1. categorising missing ethnicity as a separate category "Unknown"
2. assuming missing BMI corresponded to BMI <30 (categorised as not obese),

|  | Mortality Rate Ratio (95% CI) | | | |  |
| --- | --- | --- | --- | --- | --- |
|  | Primary Analysis* | i. Categorising missing ethnicity as "unknown" category | ii. Add assumption  that missing BMI is a BMI <30 | iii. Restricting analysis to linked population |  |
| Overall | 1.36 (1.27-1.44), p<0.0001 | 1.36 (1.28-1.45), p<0.0001 | 1.34 (1.26-1.43), p<0.0001 | 1.33 (1.25-1.42), p<0.0001 |  |
| Sex | | | | |  |
| Females | 1.36 (1.24-1.48), p<0.0001 | 1.35 (1.24-1.48), p<0.0001 | 1.33 (1.21-1.45), p<0.0001 | 1.32 (1.21-1.44), p<0.0001 |  |
| Males | 1.35 (1.24-1.48), p<0.0001 | 1.37 (1.25-1.49), p<0.0001 | 1.35 (1.23-1.47), p<0.0001 | 1.35 (1.23-1.47), p<0.0001 |  |
| Age at diagnosis | | | | |  |
| 18–29 | - | - | - | - |  |
| 30–39 | 1.37 (0.93-2.04), p=0.11 | 1.35 (0.91-1.99), p=0.13 | 1.30 (0.89-1.90), p=0.17 | 1.30 (0.88-1.92), p=0.19 |  |
| 40–49 | - | - | - | - |  |
| 50–59 | 1.43 (1.22-1.68), p<0.0001 | 1.46 (1.24-1.71), p<0.0001 | 1.43 (1.22-1.68), p<0.0001 | 1.41 (1.21-1.66), p<0.0001 |  |
| 60–69 | 1.49 (1.30-1.71), p<0.0001 | 1.49 (1.31-1.71), p<0.0001 | 1.46 (1.28-1.67), p<0.0001 | 1.46 (1.28-1.67), p<0.0001 |  |
| 70–80 | 1.35 (1.19-1.52), p<0.0001 | 1.34 (1.19-1.52), p<0.0001 | 1.33 (1.17-1.50), p<0.0001 | 1.32 (1.17-1.50), p<0.0001 |  |
| >80 | 1.36 (1.13-1.63), p=0.0013 | 1.34 (1.11-1.61), p=0.0021 | 1.31 (1.08-1.58), p=0.0051 | 1.34 (1.11-1.62), p=0.0023 |  |

1. restricting to cases and matched controls with linked data (reducing missing IMD and limiting analyses to individuals with linked ONS mortality data)

*The primary analysis was adjusted for adjusted for age and sex, ethnicity, year of exposure start, smoking status and comorbidity at index date (obesity, diabetes, hypertension, chronic obstructive pulmonary disease, ischaemic heart disease, chronic kidney disease, congestive cardiac failure, stroke/transient ischaemic attack and cancer).
